# A Role for Bottom-Up Synthetic Cells in the Internet of Bio-Nano Things?

**DOI:** 10.3390/molecules28145564

**Published:** 2023-07-21

**Authors:** Pasquale Stano, Pier Luigi Gentili, Luisa Damiano, Maurizio Magarini

**Affiliations:** 1 Department of Biological and Environmental Sciences and Technologies (DiSTeBA), University of Salento, 73100 Lecce, Italy; 2 Dipartimento di Chimica, Biologia e Biotecnologie, Università degli Studi di Perugia, 06123 Perugia, Italy; pierluigi.gentili@unipg.it; 3 Department of Communication, Arts and Media, IULM University, 20143 Milan, Italy; luisa.damiano@iulm.it; 4 Department of Electronics, Information and Bioengineering, Politecnico di Milano, 20133 Milan, Italy; maurizio.magarini@polimi.it

**Keywords:** internet of bio-nano things, molecular communications, neuromorphic engineering, synthetic biology, synthetic cells, chemical social robotics

## Abstract

The potential role of bottom-up Synthetic Cells (SCs) in the Internet of Bio-Nano Things (IoBNT) is discussed. In particular, this perspective paper focuses on the growing interest in networks of biological and/or artificial objects at the micro- and nanoscale (cells and subcellular parts, microelectrodes, microvessels, etc.), whereby communication takes place in an unconventional manner, i.e., via chemical signaling. The resulting “molecular communication” (MC) scenario paves the way to the development of innovative technologies that have the potential to impact biotechnology, nanomedicine, and related fields. The scenario that relies on the interconnection of natural and artificial entities is briefly introduced, highlighting how Synthetic Biology (SB) plays a central role. SB allows the construction of various types of SCs that can be designed, tailored, and programmed according to specific predefined requirements. In particular, “bottom-up” SCs are briefly described by commenting on the principles of their design and fabrication and their features (in particular, the capacity to exchange chemicals with other SCs or with natural biological cells). Although bottom-up SCs still have low complexity and thus basic functionalities, here, we introduce their potential role in the IoBNT. This perspective paper aims to stimulate interest in and discussion on the presented topics. The article also includes commentaries on MC, semantic information, minimal cognition, wetware neuromorphic engineering, and chemical social robotics, with the specific potential they can bring to the IoBNT.

## 1. Introduction

**Figure 1:**
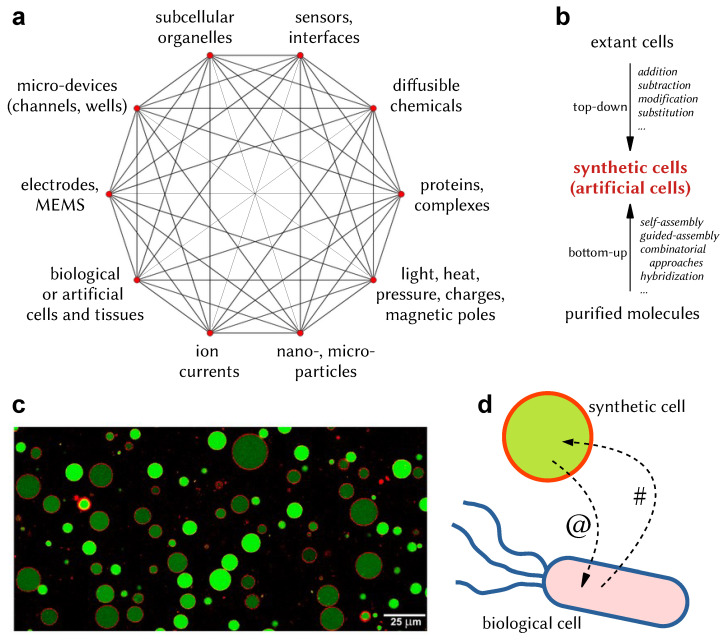
Elements of IoBNT and Synthetic Cells (SCs). (**a**) As schematically shown, the IoBNT includes bio-nanoelements that communicate with each other and realize a network that can generate complex behaviors, dynamical patterns, and phenomenologies for useful purposes. (**b**) Synthetic Cells (or artificial cells) are human-made systems that can be constructed according to two different Synthetic Biology approaches: top-down (modification of extant cells, e.g., microorganisms) and bottom-up (assembly of molecules to form cell-like structures). (**c**) Typical appearance of bottom-up SCs based on giant liposomes. Each circular object is a cross-section of a spherical microcompartment filled with a green fluorescent dye. The membrane of the compartment hosts a red fluorescent dye. Size bar represents 25 μm. The image is reproduced from [9] under the terms and conditions of the Creative Commons by Attribution (CC-BY) license. (**d**) Bottom-up SCs capable of communicating with biological cells are relevant elements of the IoBNT: schematic representation of a chemical dialogue between a synthetic cell and a bacterium, whereby the bacterium sends the chemical signal # to a SC, and the latter replies with the chemical signal @ [10]; see Section 6 for details.

The term Internet of Things (IoT), first proposed by Kevin Ashton in 1999, refers to wireless communication technologies integrated into macroscopic communication objects, i.e., “things” (computers, instruments, robots, AI systems, equipment), for tracking and monitoring operations and production lines, as well as managing infrastructures and distribution chains. While the transition toward the IoT is progressing fairly well, fueled by several technological advancements in microelectronics and optical or radiofrequency signals, a new ambitious scenario has been already put forward, driven instead by the progress in biosciences and biotechnologies. The Internet of Bio-Nano Things (IoBNT) [1,2,3]—certainly an evocative term—focuses on the interaction between nano- or microscale biological or artificial objects (parts, devices, systems) that communicate with each other in an unconventional manner to realize a bio-nanonetwork of elements designed to achieve specific functions or to behave in a coordinated manner. In particular, the IoBNT is a scenario whereby bio-nano objects (things) communicate at the nanoscale in an unconventional manner, i.e., mainly through chemical exchanges, realizing a new technology based on molecular communication. Biological and artificial objects (parts, devices, and systems, typically constructed via nanotechnology and Synthetic Biology) are interfaced with each other, generating complex behaviors, patterns, and phenomenologies for useful purposes. The elements of the IoBNT are bio-nanosensors, engineered cells, and several sorts of bio-nanodevices (Figure 1a). A novel aspect that is characteristic of the IoBNT is the exploitation of synthetic biodevices or electronic devices when interfaced with each other or with natural biological elements.

The communication and networking requirements of IoBNT applications are evident. As mentioned above, the type of communication exploited in the IoBNT is unconventional when compared to traditional communication devices: it mostly belongs to the *chemical or physicochemical domain*. Communication engineers, indeed, are currently involved in developing a new engineering branch, known as “Molecular Communication” (MC) [4,5,6], where the fundamental modality of communication is the exchange of chemicals to encode and transfer information. The related theories and implementations need to be developed essentially from scratch, and advantages and limitations are emerging from current research studies, as these approaches are rather challenging. On the other hand, we foresee that interest and attractiveness will increase in the coming years because of the potentialities and the novelty that these approaches imply [7] for future biotechnologies. Similar considerations can be extended to the field of molecular computation, often discussed and recognized with the terms “unconventional computing”, “natural computing”, and similar names.

Besides nanotechnology and micro/nanoelectronics, we identify Synthetic Biology (SB) as the best candidate companion to the IoBNT enterprise. SB is a relatively young discipline at the intersection between engineering and biology, and it aims at constructing artificial biological parts, devices, and systems that do not exist in nature by applying an engineering approach. Chemical signaling has been largely explored in SB systems, but most (if not all) studies refer to proof-of-principle experiments, whereby communication is demonstrated, while minor interest has been devoted to technical communication rules, which instead engage the attention of communication engineers. The distance in terms of goals, languages, and interests between the SB and MC communities is a gap that needs to be filled by promoting collaborative research and synergies [8].

In this perspective article, we intend to contribute to the nascent IoBNT scenario and to its supporting MC and SB research fields by presenting the notion of bottom-up Synthetic Cells (SCs) to the community of scholars interested in MC and the IoBNT. At the same time, the article aims to let synthetic biologists become more familiar with MC and the IoBNT, even if the latter might appear—perhaps—a bit visionary to date. However, we believe that the IoBNT can become a driving force for the development of bio-nanotechnologies with a relevant impact on human daily life in the future, and therefore, we consider the present discussion a timely contribution to the scientific community.

Moreover, we would like to take this opportunity to present a related set of approaches that have recently been developed in the chemical AI domain, namely, *neuromorphic systems*. These are neural surrogates and artificial neural networks (or neural-network-like systems) based on the computing capability of dynamical chemical systems. Their implementation from an IoBNT perspective has rarely been discussed, despite their potentialities. Finally, we will also direct attention to preliminary theoretical considerations stemming from the current research in social robotics by drawing an analogy between the (macroscopic) “human/robot” and “human/AI” scenarios and the (microscopic) “biological cells/SCs” one, which is implicit in the IoBNT. When considered from a wider viewpoint, indeed, the topics of communication, networking, collective properties, and reciprocal adaptation inevitably lead to the recruitment of concepts from cognitive sciences, artificial perception, and ecological interactions—further enriching the experimental approaches with relevant theoretical implications.

This article is organized as follows: Section 2 is an introduction to MC, while in Section 3, more details on the IoBNT are given. Section 4 is a presentation of SB and of its more ambitious goal: the construction of SCs. Section 5 reports on the principles, some technical details, and the main features of bottom-up SCs. The objective of these first four sections is to provide an introductory text for non-specialists. The central themes of this perspective article are instead developed in Section 6 and Section 7, where the features of communicating SCs and their potential role in the IoBNT are briefly illustrated, respectively. Finally, comments on neuromorphic bioengineering (Section 8) and the analogy between social robotics and the involvement of SCs in the IoBNT (Section 9) conclude the article. In the final remarks, we wrap-up the presented ideas, briefly mentioning the bioethical and safety issues that will be linked to these new technologies when fully developed.

## 2. Molecular Communications: History, Perspectives, and Challenges of a Frontier Engineering Approach

**Figure 2:**
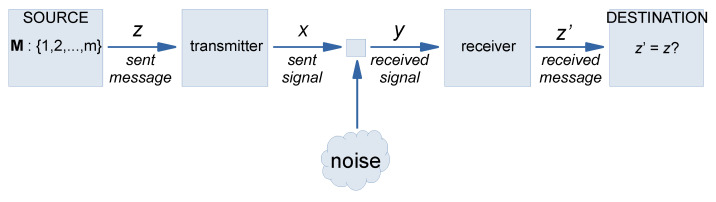
Abstract model of any communication system, taken from [5]. The source is made of a set of *m* possible messages (1, 2, …, m), one of which (e.g., the message *z*) is selected and transmitted in the form of signal *x*, which propagates through the communication channel (physically, it corresponds to the mechanisms of information transfer). Signal *y* is received, from which a received message $z^{\prime }$ is inferred. Communication is successful if, despite the noise, $z^{\prime }=z$. Note the difference between the “message” (which is generally a meaningful state of affairs for the sender and/or the receiver) and “signal” (the physical vehicle: a chemical species, a concentration gradient, etc.).

The concept of molecular communication (MC) was proposed in 2005 as an unexplored research area at the intersection of computer networks and biology. Based on the observation that biological entities such as cells communicate by using molecules, the pioneers of this field—at the time (early 2000s) with the University of California, Irvine (UCI)—proposed the idea of molecular communication, where bio-nanomachines communicate by sending and receiving information-encoded molecules in an aqueous environment [6]. In his historical retrospective article, Tadashi Nakano, one of the main proponents in the field, specifically mentions that MC ideas originated from attempts to identify a paradigm shift in communication engineering, whereby young researchers were stimulated to focus on ideas, not on the problems that reside in their subsequent implementation. In their first two articles [11,12], the MC proponents presented key concepts, terminology, and the general framework of bio-nano senders and receivers—where these bio-nano entities communicate through the five processes of encoding, sending, propagating, receiving, and decoding (Figure 2). Scenarios based on diffusion, molecular motors, and cell design were presented, together with the challenges associated with how information can be encoded/decoded in the form of molecules, fluxes, and movements or those associated with the randomness of free molecular propagation in environments. It is perhaps a sign of the *Zeitgeist* that, around the same time, Cronin and collaborators published a still-currently discussed proposal on the application of a procedure similar to the Turing test to assess the life-likeness of SCs [10,13,14], while LeDuc proposed the idea of pseudo-cell nanofactories, i.e., SCs for smart drug delivery applications [15]. In the scenarios put forward by Cronin et al. [13] and LeDuc et al. [15], indeed, the SC ability to communicate with biological cells by means of chemical exchanges is implicit (and therefore, these are paradigmatic examples of MC). The first perspective paper on communicating SCs [16] was actually inspired by the MC, Turing test, and nanofactory scenarios, attempting to integrate them in a grand vision, which is actually taking place now in contemporary SB, as mentioned in Section 6. The expansion of the MC proposal and its transformation into a sub-field of communication engineering has led to a broad community of practitioners today, with dedicated conferences, workshops, and specialized journals (e.g., *IEEE Transactions on Molecular, Biological, and Multi-Scale Communications*; *Elsevier Nano Communication Networks*; *ACM International Conference on Nanoscale Computing and Communication (ACM NanoCom)*; *Workshop on Molecular Communication (MolCom)*). Very recently, MC themes have attracted the attention of synthetic biologists [8] and experts in artificial life (i.e., the 2023 International Conference on Artificial Life, 24–28 July 2023, Sapporo-Japan, will host a workshop on MC and wetware artificial life)—hoping to combine expertise, exchange viewpoints, and attempt to combine efforts to address the challenging goals of manipulating chemical information in natural and artificial systems.

Let us briefly summarize the themes of MC, following the scholarly discussion presented in the *Molecular Communication* monograph [5]. The central theme is, of course, the extension of the human transmission of messages to the chemical domain, as it is traditionally carried out by using wired and wireless communications. While in these traditional forms of human communications, the signal is constituted by electrical voltages, currents, or electromagnetic waves, MC deals with material messages in the form of patterns of molecules—see Table 1.

The comparison between telecommunications and MC clearly shows undeniable significant differences. Despite these differences, MC engineers have been successfully working on models and simulations that can be applied to MC systems. Physico-mathematical models for MC—which are far from being trivial [17,18]—allow engineers to model communicating bio-nanomachines, including those required in hypothetical IoBNT scenarios. For example, because channel modeling occupies a pivotal role in MC, the analysis of molecular diffusion has been studied in detail. Transmitters and receivers are bio-nanomachines separated by a fluid medium, whereby signal molecules move according to Brownian motion; their spreading through the fluid can be modeled as Wiener processes. Because molecular motion is random, the arrival of transmitted molecules at the receiver is also random in time. The resulting first arrival time can be similarly simulated and analyzed in order to understand the nature of a certain specific communication channel. Modeling physical processes plays a key role in MC for performing analysis, finalizing the design, and optimizing bio-nanomachine properties for successful communication in an IoBNT scenario.

Molecular communications dominate in natural biological systems. Therefore, MC intends to realize bio-inspired communications, and this means that it aims either to engineer existing bio-communication systems or to construct biological-like ones (e.g., like in the example of communicating SCs; see Section 6). The materials used in MC systems come from the wetware world, i.e., biological and chemical materials; their size is in the meso- or nanoscale, and their functionality is, at the moment, quite basic in terms of computation capabilities (integrating two input signals, sensing one signal, etc.), even if the underlying physicochemical mechanisms can be exquisitely complex. The goal of MC, as said, is the purposeful manipulation of chemical signals to convey a message by means of bio-nanomachines, and this is realized thanks to patterns of molecules (e.g., number of molecules, type of molecules, molecular transmission timing) [18]. The propagation of molecules typically occurs by diffusion: this mechanism is a remarkable source of constraints (slow rate) or distortion (when the message is delivered in the form of a small number of molecules, Brownian motion implies that molecules can be lost or arrive at the receiver with a considerable delay) [19].

On the other hand, modeling MC in the physical domain is not enough, and these studies must be flanked by information-theoretical analyses, particularly those that are generally understood in terms of the amount of information that can be transmitted in a communication channel *à la* Shannon (Figure 2).

Here, the interaction between sender and receiver bio-nanosystems is analyzed for statistical correlations. The randomness present in all steps pertinent to the MC system increases the uncertainty about the message, i.e., the conditional probability of observing a certain channel output *y* given a channel input *x*. This will be related to the characteristic of the physical layer of the MC system, but—in general—it can be understood by applying the standard information and communication theory [20]. Many advancements have been reported, which can be recognized as dealing with “syntactic information”, e.g., the information content of a source expressed in bits per emitted symbol, which relies on the concept of Shannon entropy, or the reliable transfer of information, which, in turn, is based on the concept of channel capacity and its maximization. The Shannon capacity is obtained from the maximization of the mutual information *I*, which is a measure of the mutual dependence between the random variables that describe the source and destination elements in an MC channel. Interesting results have been obtained, helping to define the MC events in a rigorous and quantitative mathematical framework [19,21,22,23,24].

Attempts to adapt the Shannon information and communication theory [20] to the specific nature of MC are, therefore, rather recent. In this respect, early reports on the application of information theory to biomolecular interactions have just a historical connotation [25]. The sought theory needs to develop information-theoretical equivalents for the biochemical components of the model (transmitter, receiver, channel), as is generally implemented for traditional wired and wireless communications. Key terms such as channel modeling, noise analysis, information-theoretical capacity of MC, simulations and model analysis for optimization purposes, system-level research, and swarm systems are now under in-depth scrutiny by MC engineers, whose background is mainly theoretically and simulation-based. Experiments, however, are also in the MC agenda and under continuous improvement. It should be added that the intrinsic multidisciplinary facet of MC is greatly advantaged by collaborative activities among specialists in different fields, as advocated by [8].

## 3. Internet of Bio-Nano Things (IoBNT)

The IoBNT, as mentioned above, is conceived as a network of nanoscale and biological devices communicating by unconventional means—via chemical exchanges—and operating in a biological environment, e.g., the human body. Bio-nano things interfacing with each other and with micro- and nanodevices, not necessarily of a biochemical nature, might be considered an Intranet (with respect to the biological body in which they are located). However, IoBNT proponents have also imagined the use of proper bio-cyber interfaces to connect these bio-nanonetworks with the outer environment and, ultimately, with the Internet. This, in the words of these authors, is “to deliver intra-body status parameters (and receive commands and instructions) to (from) a healthcare provider” [1]. To realize IoBNT goals, two key abilities can be identified: (i) computing with bio-nano things and exploiting the related communication abilities thanks to the unique feature of operating across bio/chemical interfaces; (ii) inventing new modalities for managing chemical signals, i.e., developing new versions of Information and Communication Technologies (ICTs) in the bio/chemical domain, i.e., bio/chem-ICTs [4,16,26,27]. The main challenge that scientists and engineers need to face in the IoBNT scenario is the control of molecular communications despite their somewhat evasive behavior and often complex nature (nonlinear, cooperative, stochastic, multiscale), which become especially evident when compared to accurate communications based on the optical, electrical, and electronic devices largely employed so far (see Table 1). In the IoBNT scenario, the camp is not populated by macroscopic objects and processes but rather by micro- and nanoscopic objects and bio/chemical events, particularly those characterized by granulated matter, specific yet stochastic interactions, low energy consumption, random motion, intrinsic parallelism, and, often, full biocompatibility.

The IoBNT is thus a composite and complex scenario that requires the integration of new and traditional concepts and techniques, and it should be conceived from the medium-/long-term perspective as a new technology that will change the way we understand and exploit processes in chemical and biological contexts. Bio-nanoscale processes are indeed at the roots of biotechnology for the agri-food and marine industries, pharmaceutics, health and medicine, and even environmental and ecosystem protection. One of the major applications of the IoBNT will probably refer to medicine, pharmacology, and diagnostics, which are obviously relevant for practical reasons. Drug delivery and cellular diagnostics can be considered of primary interest, together with intracellular therapy and tissue bioengineering (regeneration control and tissue healing) [5]. Industrial and environmental applications are arenas for the IoBNT as well. The production of biomaterials can be affected by systems capable of radically modifying or controlling (bio)chemical processes, with the consequence that the so-achieved novel molecular structures and scaffolds can give rise to emergent properties in the macroscopic material. The increased risk of environmental breakdown requires better monitoring activities, typically based on sensor networks. The amplification of chemical traces can be advantageous in terms of timely interventions. Superior sensing features will be possible when biological and artificial components can communicate smoothly and efficiently at the nanoscale. Even looking at the concise and descriptive summary of applications taken from [5] reveals that networking bio-nano things of biological or artificial origin can give rise to interesting technology that enables activities going beyond the capabilities of traditional technologies [2]. We can recognize the IoBNT “embryos” in small networks of biological systems interfaced with artificial devices: micro-electromechanical systems (MEMS), biosensors, and lab-on-a-chip technologies [18,28].

We believe, moreover, that IoBNT scenarios with innovative character will definitely come from interfacing living systems (living cells) with SCs—as will be argued in Section 4 and Section 5. The successful operations between bio-nanodevices will strongly depend on how they communicate with their environment or with each other: that is, on how they sense or perceive their surroundings. The behavior of artificial bio-nanodevices can range from a simple stimulus response (automatic) to more complex autonomous (or semi-autonomous) patterns [29] (“autonomatic” [30]). Ultimately, artificial bio-nanodevices must be designed in order to selectively sense or perceive signals that “make sense” to them. By means of these elementary, yet crucial, communicative acts, bio-nanodevices will coordinate their individual or collective actions (e.g., synchronize their behavior or create organized patterns) to elicit the occurrence of complex functions. A related goal deals with the way in which they will be designed and optimized to display advanced features, such as perception and cognition [31,32]. The futuristic scenario of brain–machine interfaces also relies on the development of IoBNT technologies.

## 4. Synthetic Biology and Synthetic Cells

It is often mentioned that the name Synthetic Biology was coined either more than hundred years ago [33] or in the 1970s [34,35]. However, it is only thanks to recent advancements in genetic manipulation, bioengineering, and mathematical modeling that SB has flourished as a fascinating frontier research field that looks at basic and applied research. In the first vein (basic research), we can identify fundamental questions to which SB approaches can provide possible answers: What is the minimal complexity for life? Is it possible to construct a living cell from scratch? Can the synthetic approach help in understanding how cellular life emerged on Earth? Or epistemological questions may even be explored: for example, what kind of scientific knowledge is generated by the synthetic method employed in SB? A characteristic of this kind of SB is that its methods are used to deal with questions like those listed above, and others, by constructing appropriate structures and models, very much like a chemist would do when exploring the relations between the molecular structure and properties of materials (the definition “chemical SB” has sometimes been used to refer to such SB approaches [36,37]). In other words, SB models actually embody certain aspects of a theory of reference, which is tested experimentally. On the other hand, more practical purposes characterize SB too. These are industrial issues, including the production of specific compounds from microorganisms (bio-fuel, drugs, high-value compounds for the chemical or pharmaceutical industry, etc.), the use of microorganisms for non-medical applications (bioremediation, fermentation, hybrid living–organic or living–inorganic material, biosensing, etc.), or the application of cell-free technology for research and bioproduction [38,39,40]. Relevant SB applications in medicine focus on developing artificial biosystems for attacking cancer cells or infectious bacteria, virus, or fungi (or for diagnostics, etc.). It is supposed that SB approaches for biomedical applications will take advantage, or—better—will be crucial elements, of the above-mentioned IoBNT scenarios [1,2].

A relevant goal in SB is the construction of SCs. “Artificial Cells” (ACs) can be considered to be synonymous with SCs, even though the two adjectives (artificial and synthetic) have slightly different nuances of meaning. Moreover, it should be added that the terms “top-down” and “bottom-up”, already introduced in Figure 1 and discussed below, can be misleading too. For commentaries, see [41,42] and Damiano and Stano (manuscript in preparation). A formal definition of SCs would find its place here. It can be said, generally speaking, that SCs are cell-like structures constructed in the laboratory, having variable complexity. Indeed, this field is experiencing the liberty and the creativity that are typical of new research areas, and the name SCs, de facto, is a kind of umbrella-term for related but non-identical approaches. Defining an exact SC taxonomy is particularly difficult, because SCs, to date, have been built for different purposes, with different materials, and by scholars interested in different questions, and the achieved results have been presented according to different perspectives. Actually, epistemological studies on the very question of *what SCs are* are rare [42,43,44,45,46]. A common classification is based on the so-called top-down or bottom-up approaches [47], briefly illustrated in Figure 1b. By a top-down approach to SCs, we mean a bioengineering approach devoted to modifying extant organisms (bacteria, yeast, plants, etc.) via system-level interventions, such as rewiring the metabolic, sensorial, or genetic-control networks [48,49], or by adding, deleting, modifying, inserting, etc., novel bioelements in the systems (new proteins, new genes, new regulators, etc.). Whole-genome transplantation is the most radical manner of rewiring a cell [50]. An artificial genome, which can be designed ad hoc and be constructed by a combination of chemical and biological methods, can be introduced into living cells, where it takes control. Aiming at constructing living SCs of minimal complexity (minimal SCs hosting a minimal genome), a new species was generated by using this approach, called Synthia (*Mycoplasma mycoides* JCVI-syn1.0 [51], later optimized and improved [52,53,54,55]). It is possible to imagine that such an approach or its variants could lead to SCs matching specific behavioral requirements. While it should be recognized that the living SCs obtained by the top-down approach can be versatile and complex (i.e., functionally rich) elements of the IoBNT, the present paper is not devoted to them. Rather, we will focus on SCs that are built according to the alternative path, namely, the bottom-up one, as discussed in Section 5. Bottom-up SCs are cell-like structures literally built from scratch, starting from simple materials such as nucleic acids, enzymes, ribosomes, lipids, salts, small metabolites, etc. A final remark on SB and its underlying philosophy is required. By assuming an engineering perspective, SB has generated the concept of standard biological parts (also referred to as *biobricks*; see https://parts.igem.org/, accessed on 20 July 2023) as an analogy to standard electronic parts used in electronic engineering [56,57]. Standard biological parts (in particular, genetic sequences such as promoter regions, open reading frames, primers, plasmids, ribosomal binding sites, terminators, etc., but also proteins classified as reporters, inverters, receptors, senders, etc.) are categorized in a rigorous manner and allow the facile design and construction of SB parts, devices, and systems. In addition to the concept of standardization, concepts borrowed from engineering are largely employed in SB, such as optimization cycles (design–build–test–learn), modularization (creation of specialized molecular subsystems that perform specific functions), and orthogonalization (eliminating between-part or between-module interference). The intensive use of mathematical models for the validation and correction of new genetic or metabolic circuits often accompanies, i.e., precedes and follows, experimental efforts. Although these concepts, tools, and terms are typically employed in top-down approaches (engineered cells), they are used in bottom-up approaches too.

## 5. Bottom-Up SCs: Principles, Construction, and Features

**Figure 3:**
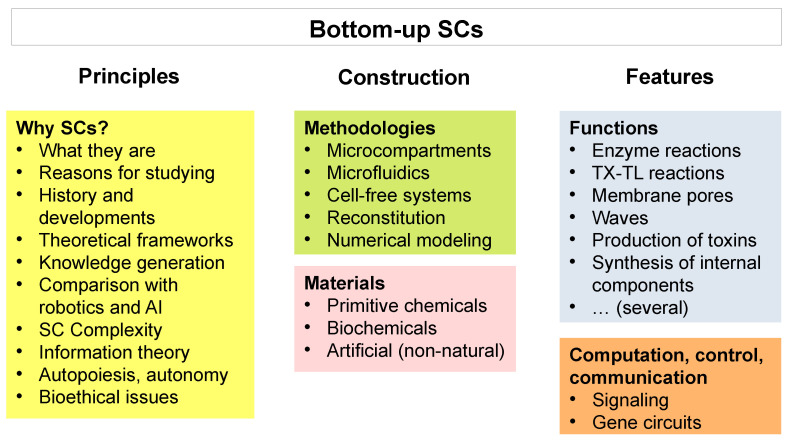
Schematic representation of some of the principles, construction methods, and features of bottom-up SCs. Details in the text.

In this section, we will briefly review the principles, the technology, and the features of bottom-up SCs, aiming at introducing the subject to non-specialized readers (see Figure 3). Describing the technical progress in the field in detail is not within the scope of this paper (for interested readers, see [58,59,60,61,62,63,64,65,66,67,68,69,70,71,72]).

*Principles*. As mentioned above, definitions can be difficult to formulate in young, non-crystallized subjects like that of SC research. Bottom-up SCs are cell-like structures, not necessarily displaying all features of cells, that are constructed by co-encapsulating chemical or biochemical elements in a microcompartment, which is itself made of chemical or biochemical compounds. This is a rather inclusive and loose definition that just depicts the current use of terms such as SCs (or artificial cells or protocells) in most publications. It might be argued that the definition of “SCs” is possibly misleading, as it evokes the concept of cells, intended as living cells. To date, however, bottom-up SCs are *not alive*, and in most cases, they look like more or less complicated bioreactors based on some kind of microcompartment (liposomes, coacervates, polymersomes, droplets, and similar structures). This is a dutiful observation, because confusion often arises about the actual complexity and life-likeness of SCs when the topic is presented to non-specialists. In contrast to top-down SCs, which are alive and have been made by engineering biological cells to different extents, current bottom-up SC technology does not yet allow the construction of *living* SCs [14]. The ultimate goal of bottom-up research on SCs is the construction of living SCs, but it should be understood that even if this goal is achieved, the resulting SCs will be highly simplified structures compared to biological cells or to top-down SCs, and their aliveness will be measured and determined according to minimalist definitions of life. On the other hand, the simple structures of bottom-up SCs do not diminish their potential role in science and technology, but rather, it can be advantageous in some respects (safety, bioethical issues, easy production and manipulation, stability, storage, etc.). The unique combination of size, confinement, molecular retention, and the resulting self-generated individuality, semi-permeability, and dynamical and functional properties of intra-compartment reactions (organized as a network) make SCs very attractive systems in science and technology. They can currently be conceived as programmable (machine-like) systems that perform specific tasks or as useful tools for reconstructing biological networks while keeping complexity to a minimum in order to understand the network dynamics to the maximum extent. In the long run, however, higher degrees of autonomy and agency will make bottom-up SCs more like organisms than machines [29,43,73,74]. Together with the engineering principles, it should be mentioned that the practice of constructing SCs involves considerations referring to theoretical-biology issues, particularly to (i) the theory of *autopoiesis* [75,76,77,78]—with its double implications for life and cognition [79,80]; (ii) the epistemology of *synthetic methods* for generating scientific knowledge [43,44,46,81,82,83,84,85,86]; (iii) the *origin of life*, because SCs can be designed and constructed to be models of primitive cells [87,88,89,90]. The versatility of bottom-up SCs brings about a wide range of interests and possible uses. In Table 2, we summarize some scopes of SCs and similar microcompartments in several research areas of interest. These areas include many other research themes and approaches, not limited to cell-like ones. Not surprisingly, the bottom-up SB approach can generate very original proposals when the many possibilities of different designs are considered. We and others have commented in previous studies on the exciting momentum that this research has recently been experiencing [63,69,71,74,91,92,93].

*Construction*. We can consider SC technology to be a combination of three fields: (i) microcompartment technology, including microfluidics—with a particular emphasis on liposomes because of their similarity to biological cells; (ii) cell-free systems; (iii) numerical modeling. The practice of building and investigating SCs has relevant multidisciplinary traits, as it relies on chemistry, biochemistry, molecular biology, biophysics, and systems thinking.

(i) Microcompartments are crucial ingredients of SC technology. Systems such as lipid vesicles (liposomes), fatty acid vesicles, polymer vesicles, coacervates, droplets, biphasic systems, etc., have been explored. Solute-filled microcompartments are often constructed via a combination of guided-assembly and self-assembly, and the resulting structures imitate biological cells in a simplified manner. The dimensions of the microcompartments correspond to the dimensions of biological entities such as viruses, bacteria, or eukaryotic cells. The different types of compartments greatly differ in their specific properties and in their resemblance to biological cells. Liposome technology should be mentioned in more detail, because liposome-based SCs, better than other types of compartments, closely resemble biological cells. Moreover, the preparation and handling methodologies of liposomes (the so-called liposome technology [94,95,96]), developed in the past decades for drug delivery applications, constitute a versatile toolbox now gifted to SB. In particular, very large liposomes, the so-called “giant” ones (e.g., 5–20 μm), turn out to be very useful and are largely employed in many current investigations (Figure 1c). The success of giant liposomes as cell models [97,98,99] is due to three factors. (1) They can be directly and easily observed by conventional microscopy and are often treated as if they were cells (common laboratory techniques can be adapted to giant liposomes, e.g., centrifugation, lysis, separation, flow cytometry, fluorescence and confocal microscopy). (2) The discovery, in 2003, of an easy-to-run preparation method for giant liposomes (the droplet transfer method) has further facilitated the establishment of giant liposomes as SC models [99,100,101,102]. (3) Finally, giant liposomes and similar large microcompartments can be constructed in a reproducible manner via microfluidic devices. Microfluidic techniques increase the homogeneity of microcompartments, simplify the interpretation of results, and in some cases, allow for the analysis of individual compartments. Despite the current exploitation of giant liposomes, it should be kept in mind that, in a scenario in which SCs need to be employed inside a biological body (as the IoBNT is), smaller liposomes (the so-called “conventional” sub-micrometric liposomes, e.g., those whose diameter lies in the 100–250 nm range) need to be used in order to travel in blood vessels. Constructing complex SCs based on nano-sized liposomes is still challenging due to the difficulty in co-entrapping several different molecular components in such small volumes [62,103,104,105,106].

(ii) By cell-free systems, here we broadly mean all those molecular systems of chemical and/or biochemical origin that can realize simple or complex reaction networks in vitro, i.e., that can be routinely operated outside the cellular *milieu*. There is a broad range of possibilities, ranging from chemical to enzymatic reactions. To produce SCs, then, one just needs to encapsulate the components of the reactive cell-free system of interest inside the compartment of interest. Solute-filled compartments will be generated, while the external non-entrapped material will be removed or inhibited. The cell-free system can be made of several enzymes in order to build SCs hosting (multi)enzymatic processes, but much of the research has focused on the DNA → mRNA → protein processes (gene expression), embodying the central dogma of biology and allowing the production of proteins from the corresponding genetic DNA sequences. This procedure is more sophisticated than just inserting enzymes (or other ready-to-operate proteins, such as sensors, regulators, etc.) but confers a cell-like *organizational trait* to SCs that makes them interesting for a wider range of research questions. Cell-free systems that are competent for the latter reactions are TX-TL systems (TX: transcription; TL: translation) and can be made of cellular extracts or from reconstituted mixtures [107]; they include around one hundred components. Because the proteins are the “actuators” of biological cells and of SCs (see Table 3), endowing SCs with a TX-TL *module* paves the way to generative functionalization in situ. Moreover, gene expression can be controlled by signal molecules or by internal regulatory circuits, and the resulting SCs become, in a certain sense, programmable. Crucial elements that definitely enrich SC operations are membrane proteins and, in particular, membrane sensors or receptors, which transduce external signals to internal ones. These can be inserted into the SC when it is constructed, or the TX-TL system can be made to generate them [108,109,110,111,112]. While the production of water-soluble proteins not requiring post-translational modifications is generally seen as feasible and successful, when membrane proteins are considered, the task becomes more challenging. Consequently, this type of functionalization—which cannot be considered routinely achieved yet—is critical for truly advancing SC technology.

(iii) Numerical modeling is highly relevant in SB, especially when applied to metabolic engineering, e.g., when ad hoc modified (micro)organisms are designed for a bioproduction scope. Modeling becomes an invaluable support before and during optimization. So far, bottom-up SCs still have a low complexity level, and resorting to sophisticated mathematical models has received limited attention. However, when targeting the construction of bottom-up SCs with non-trivial genetic circuits (aiming to develop control modules; see Table 3) or signaling cascades or chemical neural networks for computation purposes [113,114], etc., the achievement of the desired pattern is best predicted by modeling [115,116,117,118,119]. On the other hand, the stochastic nature of solute encapsulation and reaction kinetics in SCs offers the opportunity for modeling the experimental results accordingly so that deviations from expected behavior can be spotted and, if interesting, further explored [120,121]. When one looks at SCs from a functional perspective, i.e., as operators capable of handling information, mathematical models that describe information flows, according to the Shannon theory of communication, become interesting too. This perspective is not included in most current studies, with rare exceptions [122,123]. However, as will be commented on in Section 6.1, this turn toward information theory can become very productive, because it matches the IoBNT framework (syntactic information), and moreover, according to some recent approaches [123,124], it can provide insights about semantic information [125]. Interestingly, these approaches can contribute to deciphering long-standing issues, such as autonomy, agency [124,126,127,128,129], and the generation of meaning [130]. In this regard, theoretically oriented SCs represent an invaluable experimental platform to face these issues in a very innovative manner [29,31,32,131].

*Features*. What makes the idea of constructing SCs from scratch attractive is the potential for full design and programmability. In a sense, SCs can be conceived as the wetware implementation of artificial systems [81,83,86], to be placed side by side with software and hardware ones (e.g., AI algorithms and robots, respectively; see Table 2). This feature is certainly fascinating and relates to the human desire and ability to fabricate artificial systems that simulate biological ones, in terms of being alive and intelligent—somewhat resonating with the ancient Prometheus myth. To date, however, the extent to which SCs really imitate biological life or are programmable like a computer or a robot is still an open question, amenable to discussion and experimental verification. It can be said that considering how well current SC technology is advancing, a generalized optimism about achieving systems with a certain degree of complexity and functionalization is favorably reckoned. The exciting momentum of contemporary SC research has not appeared suddenly but is a result of a path started in the early 1990 when the first attempts at chemical implementations of autopoiesis were carried out [87,132,133], together with the very idea of constructing SCs by means of liposomes, in the context of origin-of-life studies—a practice that has since flowed into SB and systems chemistry [134,135].

Several functions have been reconstructed and implemented in SCs. Some refer to chemical transformations, others correspond to information flows, and still others relate to energetic autonomy, or self-reproduction. SCs then become competent for enzyme reactions, gene expression (as specified above), metabolic pathways for lipid synthesis [136], simple forms of control based on the activation of gene expression, the enzymatic production of signal molecules, sensing signal molecules, the formation of pores and gap junctions [137,138] in the membrane and periodic reaction–diffusion waves [139], genetic material replication, and the production of toxins [140,141,142]. Additional relevant functions, like bio-energy generation, have recently been achieved [105,143,144,145], while others are under preliminary analyses (e.g., reducing power for metabolism [146]; chemical neural networks for computation and control [114]). These functions have generally been implemented individually, one at a time. However, in order to advance SC technology, efforts should be directed at their co-implementation in the same SC. It is worth noting, then, that to progress toward more sophisticated systems, non-trivial stages of optimization and integration should be completed with success. It has been proposed that such ambitious goals are best approached by using evolutionary strategies [64].

Despite these challenging and sometimes not-yet-reached goals, the general idea is that complex SCs can be designed in terms of “modules”, defined as sub-systems carrying out specific functions. Enzymes (proteins) are the SC workhorses (i.e., sensors, actuators, controllers) and their presence in SCs can either be included at the time of SC construction or be generated in situ by installed ribosomal machinery and thus ultimately determined by a DNA (or mRNA) sequence. In the latter case, the TX-TL machinery is a sort of *chassis*, wherein specific genetic programs can be plugged in. At the same time, however, to achieve more complex systems (autonomous, controllable, programmable), the chassis needs to be complemented with pre-fabricated modules devoted to specialized functions (sensing, energy production, exporting, etc.). In other words, SC features can be tuned depending on the specific target applications; approaches and strategies can be blended together in an opportunistic manner. This utilitarian strategy is particularly efficient in goal-driven designs. SCs that address more theoretical issues should be necessarily based on mechanisms compatible with the constraints imposed by the theory under examination/exploration [45].

Among the various behaviors and features that one could, in principle, install in SCs, certainly the capacity to communicate acquires the utmost importance, and it will be commented on more extensively in Section 6. Communicating SCs are now a hot topic in bottom-up SB, and several systems have been investigated and reported. Table 3 summarizes some modules and functions that might have high relevance for SCs from an IoBNT perspective.

**Table 3:** SC features that become relevant from MC and IoBNT perspectives.

Module	Specifications/Possible Realization	Notes
Sensor	Receptors on the surface and in the lumen	The sensor array selectively (and explicitly) determines which factors the SC will respond to. However, because isolating molecular interactions is not possible, in principle, any SC element can be de facto influenced by third-party molecules.
Actuator	Any element or module that can modify the SC dynamics, either to assure SC maintenance, self-distinction, and existence or to achieve a useful programmed goal	It must be activated upon computation.
Control, Computation	Switch-like mechanisms, triggers, activation, transistor-like elements [147], chemical neural networks	This should not necessarily be a genetic mechanism; protein functions are often regulated allosterically, and cooperative mechanisms originate a sigmoidal response, signal integration, input classification, recurrence, and self-stabilization.
Communication	Actually a combination of sensors, controllers, actuators; explicitly, a module that imitates biological communication mechanisms. More generally, any physicochemical mechanism interpreted as an information transfer device	Examples: covering short-, medium-, and long-range distances (e.g., Ca${}^{2+}$, molecular motors on filaments, diffusion-driven signaling).
Power/Energy	Substrate-level phosphorylation, photo-phosphorylation	The ultimate source of energy must be able to enter the SC, i.e., to be recruited by and to participate in the chemical organization/network.

## 6. Engagement of Bottom-Up SCs and Alike Systems in Molecular Communication

The innovative perspective of MC is based on the analogy between hardware components, such as sensors, controllers, and actuators [148], and their molecular counterparts. When SCs operate by gene expression, sensors can be protein receptors or RNA aptamers that bind to a signal molecule and consequently change its conformation. This event directly or indirectly affects SC modules dedicated to gene expression. Indeed, these are mechanisms of the regulation of gene expression by protein receptors or RNA aptamers (riboswitches) at the transcriptional or translational level, respectively. The production of proteins, for instance, can be promoted or inhibited upon receiving a message in the form of a chemical compound. Genetic circuits of distinctive complexity can behave in a highly specific manner, leading to the activation or inhibition of gene transcription. The end-point of these intra-SC processes (the in situ production of proteins) indeed constitutes the major element of interest. By regulating intra-SC protein synthesis, signals can control SC functions. Proteins are actuators and can perform several operations, including operations such as signal production or signal revealing. In this manner, it is possible to construct sender, receiver, or sender-and-receiver SCs—as in the renowned report by Mansy and collaborators [10] (see Figure 1d). Experimental results based on SCs that produce or sense bacterial signaling molecules, such as acyl-homoserine lactones, have been reported [16,149], as well as approaches to exchanging chemicals between SCs and biological cells in close contact via connexin [138] (interestingly, SCs synthesized connexin via transcription–translation). Readers interested in learning in extenso the motivations and recent experimental steps in the development of communicating SCs and alike structures can refer to the several articles published so far [10,16,66,122,141,149,150,151,152,153,154,155,156,157,158,159,160,161,162,163] (this list is not comprehensive). As mentioned above, not only SCs but also other systems, mainly nanoparticles, have been investigated for their molecular communication capabilities with living biological cells. For example, nanoparticles having a high specific surface area also have the possibility of being functionalized with chemical moieties, DNA strands, enzymes, and antibodies [164,165]. Cascade-like linear communication [166], exchanges of molecular messengers with living microorganisms [167], and nano-translation to enable communication between different microorganisms [168] are just some of the undertaken research directions.

From a practical perspective, endowing SCs with communicative ability is seen as a prerequisite for their use as smart drug delivery systems [15,169]. For example, one can imagine SCs that are injected into the body and then travel to and extravasate in the target tissue, for example, by taking advantage of loose vascularization capillaries near solid tumors. Once there, they need to recognize the target tissue via specific antigen/antibody recognition, bind to the cells, and activate internal mechanisms upon the perception of chemical signals sent by the target tissue cells. The so-designed SCs would sense the environment and produce and release therapeutics thanks to an available, yet inactive until that moment, drug production module [15]. Alternatively, SCs loaded with drugs could be internalized in the target cells. Pioneering experiments based on simple SCs as producers of anti-tumor toxins have been reported [140].

### 6.1. MC Implications of Communicating SCs: Evaluating Syntactic and Semantic Information

As briefly explained in Section 2, a major pillar of current MC studies refers to the application—with due adaptations—of the Shannon communication theory to the realm of molecules, chemical signals, bio-nanomachines, and all aspects of nanonetworking scenarios. Communications in the IoBNT are typically (but not exclusively) based on molecular exchanges, molecular diffusion, chemical binding, and reactions. Chemical information, in turn, is embodied in the concentration of chemicals, in their structures (topologies), in spatio-temporal patterns, in self-organizing networks, or in the out-of-equilibrium states of certain chemical systems. Because of their peculiar nature, chemical signals differ significantly from electromagnetic ones (Table 1); similarly, chemical information and chemical computation differ from the corresponding concepts in conventional systems.

Current MC approaches have been largely based on syntactic information, referring to the usual connotation of Shannon information. Since its introduction, in fact, the concepts of information and the communication of information deal with the selection of a message from a set of messages in the information source. Shannon gave information on a numerical value based on probability (Shannon entropy), and it is a measure of the decrease in uncertainty for a receiver (the amount of information being inversely proportional to the probability of the occurrence of that information). However, as Shannon himself emphasized, the semantic aspects of information are irrelevant for his theory [20].

On the other hand, in addition to syntactic information, semantic information, meaning the information that makes sense to an agent [31,32,170], plays a key role in all biological organisms. Semantic aspects of information are absent in the Shannon theory but were present in the early days of cybernetics [171] and furthered, e.g., by Donald M. MacKay [125] and Gregory Bateson [172]. It has recently been put forward that semantic information deserves scientific exploration, taking advantage of novel proposals for its quantification [31]. Its relation to MC, SB, and, in general, the field of the generation and engineering of *meaning* in artificial systems seems indeed particularly interesting and suitable for current research. Moreover, besides being novel and unexplored, the approaches to semantic information refer to an issue that goes beyond the MC perimeter, as they could provide a working framework for exploring the semantic aspects of information in all kinds of artifacts (robots, AI systems, SB constructions).

An in-depth discussion about semantic information and meaning lies outside the scope of this article. However, it can be mentioned that, according to MacKay, the concept of semantic information is related to what information *does* when received by an agent [125], i.e., how it changes the internal constraints. It refers to the changes that a message causes in the internal organization of the receiver, with the consequence of modifying its current and future behavior. Seen from this perspective, the generation of meaning in the agents can be considered a further instance of agent/environment super-system self-organization. The technical definition of meaning, according to this school, is the “selective function” that “any event that can be detected by an organism or a machine may exercise […] upon the ensemble of transition probabilities of the behavior of the detector (it operates upon the statistical parameters of the system representing the detector)” [125,173]. This selective function originated in biological systems during evolution. However, from the modern SB perspective, and in the IoBNT scenario, a related question becomes interesting, i.e., whether or not it is possible to engineer the meaning of information thanks to the possibility of designing a cell-like system from scratch (or, in the case of engineered cells, thanks to the possibility of “rewiring” their genetic circuitry). In any case, dealing with semantic information means searching for the causal role that perceived information (e.g., chemical signals) plays in the recipient mechanisms, behaviors (tasks), and ultimately, its own existence.

**Figure 4:**
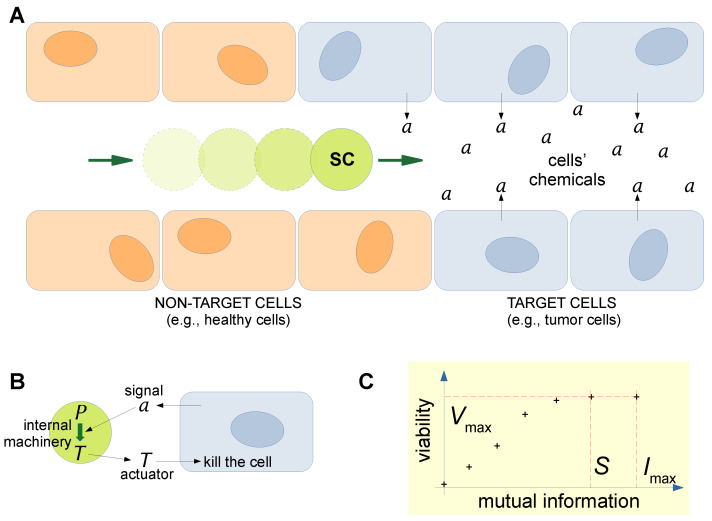
A realistic scenario of SCs as smart drug delivery systems and their possible use as a model for quantifying semantic information. (**A**) An SC—endowed with sensors, controllers, and actuators—travels in a blood vessel to reach a region with a disease. The target cells, owing to their dysfunctional metabolism, produce a disease-specific chemical *a*. (**B**) A communication channel then takes place between the dysfunctional cells and the SCs, whereby the signal *a* triggers the production, inside the SCs, of a drug/toxin *T*, which is released in order to kill (or heal) the target cells. (**C**) This smart drug delivery scenario has been analyzed in terms of semantic information, as defined by Kolchinsky and Wolpert [124]. In particular, the semantic information *S* is the part of mutual information *I* exchanged between the environment (the target cells) and the agent (the SC), which is strictly necessary not to decrease the agent “viability” $V_{max}$. In a smart drug delivery context, the viability can be defined as the capacity to complete the task of sensing *a* and producing *T* (in other contexts, viability can refer to other relevant behaviors, including SC self-maintenance). Technical details in [123,174].

It is from this last perspective that the Kolchinsky and Wolpert proposal for measuring semantic information acquires relevance in SB, SC technology, and indirectly, the IoBNT [124]. Their approach is based on the information-theoretical analysis of the fraction of mutual information that an agent (e.g., an SC) exchanges with its environment (e.g., made of other SCs, or natural cells, or other devices with which SCs can interact), which is *causally necessary* to keep it alive. As specified above (Section 4 and Section 5), the SCs that can be constructed in the laboratory are—to date—not yet alive, but the Kolchinsky–Wolpert approach can nevertheless be adapted to them by focusing on the SC’s ability to complete predefined tasks. As has been evidenced in recent studies [123,174], SCs interacting in a biological *milieu* are amenable to modeling via the Kolchinsky–Wolpert strategy in order to quantify the amount of semantic information exchanged with their environment. The investigated scenario is indeed resonant with an IoBNT one, whereby there are chemicals in the environment (composed of living cells) that constitute signals for SCs, which in turn respond to chemical stimuli by producing a toxin to kill cancerous cells (Figure 4). Semantic information (in bits) can be calculated from the overall amount of mutual information, following the strategy of *interventions* (scrambling the pattern of the environmental variables in order to determine which fraction of the mutual information can be lost without a significant loss of function). For the sake of the current discussion, it is important to remark that semantic information analyses will integrate the usual information-theoretical approaches aimed at quantifying syntactic information. Taken together, syntactic and semantic information provide a complete characterization of nanobionetworks. Adding the semantic dimension provides value to classic theory, where the synergies between IoT and AI have started showing its limitations. In this context, semantic information is broadening the applicability of the classic communication-theoretic framework to the conveying of meaning through communication systems [175].

### 6.2. Toward Minimal Cognition

From a broader viewpoint, additional considerations can emerge from the development of these systems: communicating SCs can also have value in cognitive sciences. SCs can be intended as an experimental platform for testing the minimum requirements for cognition, i.e., how, and to what extent, an artificial system becomes cognitive. It should be noted that being “reactive” (generating predefined stimulus-response dynamics, determined by a structure with no plasticity, imposed by the designer and maker of the system) is not the same as being cognitive. Being reactive and cognitive are two features that are certainly related to MC and chemical signaling but differ from each other because cognitive systems are also autonomous, in the sense that they are characterized by a set of organizationally closed processes, which autonomously determine the way in which the system responds to environmental stimuli, literally selecting those that can be accommodated in its mechanisms by *plastic adaptations*. Quoting Bitbol and Luisi, it can be said that:

“[a] cognitive structure […] selects, and retroactively alters, the *stimuli* to which it is sensitive. By this combination of choice and feed-back, the organic structure determines (in a way *moulds*) its own specific environment; and the environment in turn brings the cognitive organization to its full development. The system and the environment make one another: cognition according to Maturana and Varela is a process of co-emergence”.

(italics in the original text) [79]

Another interesting point of discussion comes instead from considering communication between SCs and biological cells as the SB analog of the famous Turing imitation game [176] (also known as the Turing test for artificial intelligence). It has been proposed to exploit chemical communications in artificial cellular systems as a playground for assaying the complexity of SCs via a Turing test [13]. For example, inspired by the Turing test arguments, Mansy and collaborators have suggested that SCs that are able to communicate bidirectionally with bacteria are about 40% life-like [10]. The application of tests of imitative relevance, like the Turing test, has been, however, criticized, as they do not assess the most critically relevant one, the organizational one [14,46].

## 7. A Role for Bottom-Up Synthetic Cells in the Internet of Bio-Nano Things?

Biological systems are actually made of networks of communicating elements and subsystems. In addition to phenomena properly recognized as communicative acts (paracrine, endocrine, juxtacrine, and autocrine signaling), chemical transformations—metabolic routes, gene expression, etc.—can be thought of as communication channels [177]. This view relates MC to molecular computing according to the following natural computing rationale: any distinguishable physicochemical state of matter and energy can be used to encode information, and every natural transformation of this state is a kind of computation. In particular, it is possible to exploit physicochemical laws to perform computations. Every physicochemical law describes a causal event, and the latter can be conceived as a computation. The causes are the inputs, the effects are the outputs, and the law governing the transformation is the algorithm of the computation (see also Section 8).

The above presentations of the IoBNT (Section 3), bottom-up SC construction (Section 5), and SC communicating capabilities (Section 6) allow us to sketch simple IoBNT scenarios based on the following three theoretical/operative premises:

- The constantly improving SC technology and, more specifically, bottom-up approaches. Although in the original IoBNT literature [1], reference was mainly made to “engineered cells” (intended as ad hoc engineered living cells, which are systems obtained by the the top-down SC branch; see Figure 1b and Section 4), our particular interest here lies in the bottom-up approach, recognizing that the corresponding functionalities are certainly inferior to those of the engineered cells. To this aim, SCs can just be seen as rather simple machines, without the need to display the peculiar life-like behaviors or especially the complexity of living cells. Technical advancements from the biochemical/molecular biology perspectives are thus fundamental to reaching these goals. We also suggest that recalling theoretical considerations based on the first and second cybernetics [126,178,179,180] and artificial/biological mirroring [81] (information processing, communication, homeostasis, feedback, autonomy, etc.) can powerfully boost SB approaches: it is possible to learn from explored topics, issues, and solutions in cybernetics and apply them to SB artifacts.
- An accompanying MC theory to model, guide, and drive the above-mentioned SC developments in order to fulfill expectations of the IoBNT scenario. MC is the framework for constructing the IoBNT, whereby one needs to develop strategies that mimic what happens in biological systems and in the (already progressed) IoT.
- Their fruitful combination in a novel, integrated, synergic tool. Elements of the IoBNT can be bio-nanosensors, engineered cells, and bottom-up SCs. The combination of these elements relies on the possibility of interfacing natural or synthetic biodevices with electronic devices, as well as natural biodevices with synthetic biodevices.

**Figure 5:**
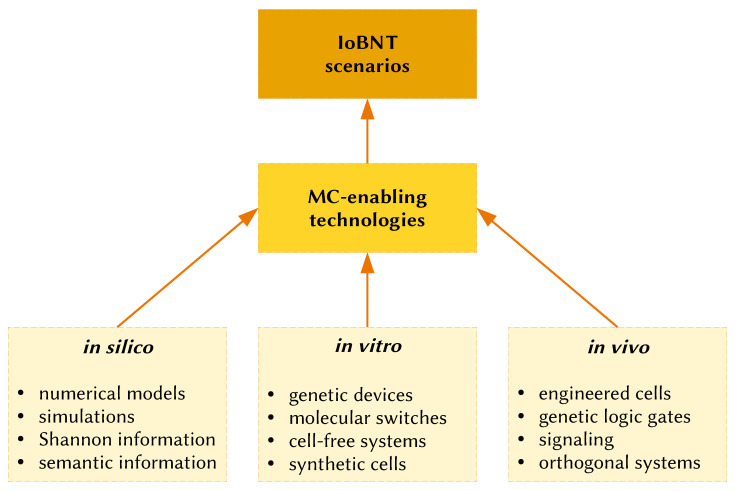
A schematic representation of SB (in all its facets: in silico, in vitro, in vivo) as a fundamental technology to develop MC to its full extent and potential, which in turn will enable the onset of IoBNT.

If the respective communities combine their efforts in a productive manner, SCs could become valuable artificial bio-nanomachines that communicate and operate through chemical signals in an IoBNT ecosystem. More generally, however, not only SCs but also the rich toolbox of structures, strategies, and approaches typically developed in SB can be considered technologies that, if developed from the MC perspective, become the building blocks of the IoBNT (Figure 5).

**Figure 6:**
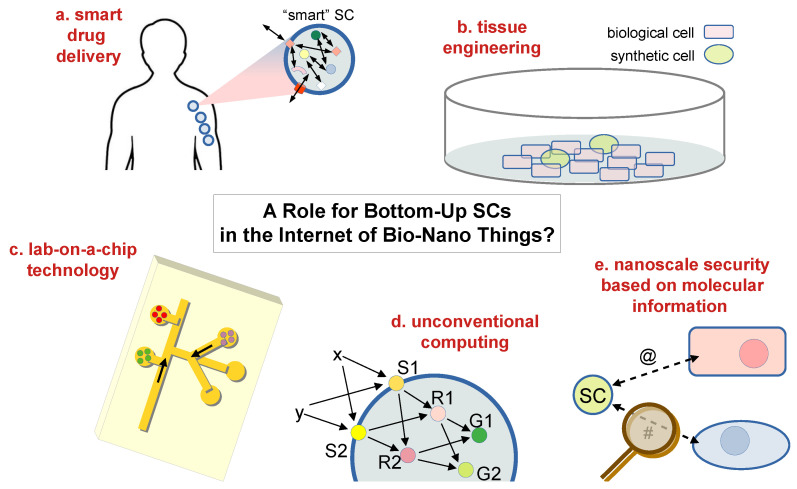
A role for bottom-up SCs in the Internet of Bio-Nano Things? Pictorial representations of possible research directions, which seem to be relevant or feasible in the short–medium term. (**a**) Smart SCs as vectors for smart drug delivery scenarios. Thanks to communicative and control features, SCs can sense their environment and produce/release a drug in a controlled and programmable manner. (**b**) Tissue engineering. An in vitro approach might benefit from MC technologies based on SCs that send/receive signals and activate internal mechanisms in order to engineer tissue development. (**c**) Lab-on-a-chip technology, microfluidic devices. Development of miniaturized (bio)chemical laboratories “on-a-chip” for assays, screening, and tests. SC features such as compartmentation, membrane-associated functionalities, biomaterial protection, semi-permeability, etc., could become essential for specific applications. (**d**) Unconventional computing. Computing with chemical species, as biological living cells do, is a scientific theme that is interesting per se, but it can also become a tool when coupled with all scenarios depicted above. Neural network architectures can be realized when SCs are designed to operate in a neuron-like manner (not shown) or at lower level, i.e., at the molecular level, when computation is performed inside SCs. In the drawing, a system inspired by bacterial two-component signaling is reported: x and y are signal molecules, S1 and S2 are sensors, R1 and R2 are response regulators, and G1 and G2 are two genes. (**e**) Nanoscale security based on molecular information. To provide innovative design and engineering techniques for future safe healthcare monitoring systems based on MC and its nanoscale constituents. In the drawing, a SC exchanges chemical signals # and @ with biological cells.

In order to discuss *realistic* roles of SCs in the IoBNT, we first need to recognize that the IoBNT describes quite complex scenarios—for any SB system and for bottom-up SCs in particular. Therefore, our discussion will consider only some selected and relevant (and possibly simple) tasks, taking inspiration from the entries sketched by MC pioneers (Chapter 8 of [5]) and commenting on their implementation by means of bottom-up SCs, according to realistic perspectives (Figure 6). The scenarios are intended to be tentative and speculative but can help put the spotlight on this in statu nascendi field.

*Drug delivery*. The development of smart drug carriers is firstly envisaged (Figure 6a), with an emphasis on the targeted and controlled delivery of drugs in order to reduce side effects. In previous sections, we have shown that SCs have already been identified as a preferential platform for developing such a technology, with a caveat, however. As mentioned above, the tremendous progress we are observing generally includes giant-liposome-based SCs (because they are easy to prepare, show high entrapment yield, and are easy to study via direct observation). However, for some medical applications (those requiring intravenous or pulmonary administrations), the particles that function as drug carriers or drug producers must be small in order to safely travel in capillaries and avoid occlusions or have a higher probability of escaping defense systems (i.e., it is common to refer to conventional liposomes, with diameters in the 100–250 nm range, as drug delivery systems). Producing SCs of that size has proved possible but much more complicated than when giant liposomes are employed [62]—as mentioned in Section 5. The other IoBNT scenario depicted in [5] refers to bio-nanomachines that perform a therapeutic act cooperatively, thanks to signal amplification, communication, and recruitment. Smart SCs can be designed, in principle, for such actions, on the basis of expected improvements in SC technology in the coming years. SCs need to be endowed with recognition, sending/receiving, and release capabilities.

*Tissue engineering*. The subject of this field is tissue/organ regeneration, again based on communicating cells. For in vitro tissue regeneration, cell culture methods are employed (Figure 6b). Scenarios have been depicted by considering that tissue growth is controlled by growth factors (cell-secreted molecules) capable of stimulating cell proliferation, wound healing, and occasionally cellular differentiation. A possibility that comes to mind is the use of SCs as sources of growth factors, in terms of a set of SCs that produce such signaling molecules in pre-determined spatio-temporal patterns (SCs at a specific location that produce and release growth factors at programmable moments). While spatial patterning does not seem too complex a goal, the timing would require a special—and certainly not trivial—design. However, because experiments with cultured cells are somewhat simpler than in vivo ones, the tissue engineering scenario can attract attention, being a platform that would provide scientific rewards. For example, a recent study reported that SCs induced endothelial cell proliferation, migration, tube formation, and angiogenesis-related intracellular signaling [181].

*Lab-on-a-chip technology*. The analytical advantages of the integration and miniaturization of lab-on-a-chip technology are well known. The reduction in the device size leads to a reduction in reagent amounts and costs; it also increases speed, efficiency, portability, and parallelization. Here, following the discussion in [5], we envisage a possible role of SCs as microcompartments/reservoirs of functional molecules for the assay (Figure 6c). If necessary, SCs can be anchored to a substrate [182]. The confinement of molecules not only allows for spatial resolution at a very low scale but also protects them against deactivation, unwanted side reactions, and diffusion. In contrast to covalent immobilization, confinement in microcompartments can preserve biofunctions for a longer time, especially when the microcompartment boundary is itself a biocompatible material (e.g., lipids in the liposome membrane).

*Unconventional computing*. Because any distinguishable physicochemical state of matter can be used to encode information, every natural transformation among different states is a kind of computation. The inputs, outputs, and algorithm of this sort of computation are the reactants (or other effectors, e.g., catalysts or modifiers), the products (or other effects caused by the reactions), and the chemical “rules”, respectively. The features of this approach to computing (also known as natural computing) should be identified, including peculiar computing architectures, high functional complexity, specificity or selectivity, and especially the massive parallelism associated with it. Unconventional computing, in general, does not compete with traditional silicon-based computing but can help with respect to specific problems, such as combinatorial ones. The IoBNT perfectly matches the architectural requirement of unconventional computing: a set of bio-nanomachines that compute and communicate with each other in a massively parallel way. An example can be a neural-network-like architecture, where instead of the silicon equivalent of a neuron, an SC is placed (similarly, unconventional computing could be realized by intra-SC chemical neural networks [29,114]; see Figure 6d). Neural-network-like dynamics is so intriguing that it has inspired a broader field—neuromorphic engineering, briefly summarized in Section 8—which we believe nicely complements the current discussion on the unconventional computing facet of the IoBNT.

*Nanoscale security based on molecular information*. To provide the groundwork for innovative design and engineering techniques for future safe healthcare monitoring systems based on MC and its nanoscale constituents (Figure 6e), these transdisciplinary strategies will draw on knowledge from existing physical layer security (PLS) techniques used at the cutting edge of the IoT and are based on how information is encoded, processed, and transferred in biological systems. An unparalleled level of access to molecular and biochemical information, particularly within and around the human body, will be made possible by the IoBNT, which aims to establish a seamless and widespread link between the network and the biological world. These networked sensing and computing systems, which rely on molecules and chemical processes rather than electronics, have security implications that have not yet been properly studied. This open question presents both a challenge and an opportunity. The following research directions are being pursued: (i) molecular information-based characterization of IoBNT security threats; (ii) data analysis to link healthcare crises to IoBNT security threats; and (iii) the development of IoBNT security solutions for healthcare administration.

To summarize, bottom-up SCs can surely play a role in the IoBNT, and this would take advantage of bottom-up SC technology. The design and the structure of bottom-up SCs are versatile, can combine biological and artificial materials quite effectively, and can be designed in order to be robust and capable of interfacing with biological and other chemical environments.

## 8. IoBNT through Neuromorphic Engineering in Wetware

The remarkable features and functionalities of living cells can be mimicked chemically through the bottom-up approach, as explained in the previous sections, and dynamically through the selection of peculiar composite chemical systems. The latter chemical systems usually possess “chemical bricks” that are quite different from those peculiar to living beings. Nonetheless, they can reproduce some cellular activities, such as self-replication, metabolism, evolutionary processes, and information handling, because they are complex nonlinear chemical systems that work very far from the thermodynamic equilibrium [183]. Some chemical systems in wetware have been proposed as surrogates of the cells specialized in handling information, i.e., the neurons.

These neural surrogates can communicate through electrochemical or optical signals [184,185,186,187,188]. Networks of neural surrogates constitute a promising approach for implementing the IoBNT, especially those exchanging optical signals. They can easily interact with the Internet cyber-domain based on electrical circuits and electromagnetic communications [1]. Neural surrogates that exchange optical signals can be organized in feedforward and recurrent neural networks, showing spontaneous phenomena of temporal self-organization [189]. When synchronization involves chaotic signals, it allows secure communications and encryption [190,191,192,193,194]. Some neural surrogates in wetware have been implemented through photochromic and luminescent molecules [194,195]. The wavelength-dependent response of photochromic compounds makes them appropriate for reproducing neuromodulation [196]. Neuromodulation is the power of reconfiguring neural networks into different functional circuits depending on the stimuli: it allows animals to learn [197]. By simply changing the radiation wavelength, it is possible to reconfigure the architecture of the IoBNT. Furthermore, opto-based IoBNTs are valuable for facing challenges in computing. They can be employed for recognizing variable patterns and proposing solutions to intractable NP problems.

Since the computational power of IoBNTs depends primarily on the number of nodes, it is of paramount importance to miniaturize them. The most promising solutions for their miniaturization are through single droplets of microemulsions [198], liposomes [199], liquid marbles [200], and nanocapsules [201]. Miniaturization can be pushed to the limit of single molecules when proteins are exploited as neural surrogates [202]. Proteins can become neural models due to their complex three-dimensional structures that rule their chemical behavior. In this regard, Intrinsically Disordered Proteins (IDPs) are particularly promising [203]. Any IDP is an abundant collection of conformers, whose composition and behavior are susceptible to the context. IDPs can help to devise IoBNTs that are able to process fuzzy logic [114,204] and make decisions in environments dominated by uncertainty, partiality, and the relativity of truth [205,206]. They can become the fundamental ingredients of chemical robots expected to interact with the natural cells and organelles of living beings to perform biomedical actions. Those chemical robots could become auxiliary elements of the natural immune system, a pristine instance of a natural internet of cells and molecules protecting living beings.

## 9. Social Robotics in the Chemical Domain: Epistemological Considerations of the Synthetic and Hybrid Ecologies Emerging from Cross-Fertilization between SC and IoBNT

One of the most interesting implications of the synergy between SCs and the IoBNT concerns the development, within the chemical domain, of the emerging techno-scientific area called *social robotics*, born at the dawn of the new millennium from the convergence of frontier fields in robotics such as *Human–Robot Interaction* (HRI), *Cognitive Robotics*, and *Social Embodied AI*, among others [207]. Since its birth, social robotics has been engaged in the design and construction of electromechanical robots capable of interacting through social signals—e.g., gaze, gestures, emotional expressions, verbal communication. The main directions of development that the field has been exploring, so far, are two. The first, organized around the notion of *robotic sociality*, is guided by the ambition to create robotic agents that communicate with each other through shared signals and, on this basis, generate robotic social aggregates endowed with novel cognitive capacities of a collective nature, as occurs in paradigmatic cases of social insects and, more generally, in social forms of collective intelligence (e.g., [208,209]). A shift in focus from robotic to hybrid forms of sociality characterizes the second and currently dominant direction of social robotics, which works toward the implementation of efficacious social interactions between natural and robotic agents. In this initial phase of its evolution, this explorative axis concentrates its efforts—almost exclusively—on creating robotic “social partners” for humans, which in the field are conceived of as robots capable of communicating with humans through social signals compatible with human ones.

The second, mainstream project, dedicated to introducing more intuitive and effective ways of interacting with robotic platforms destined for a variety of operational uses (e.g., [210]), finds its key implementation factor in the construction of a “social presence” for robots, that is, appearance and behavioral features apt to stimulate humans to recognize these machines as agents with whom they can interact socially. By creating convincing forms of robotic social presence, social robotics targets the development of mixed (human–robot) “social ecologies”, prospected as hybrid social contexts in which social collaboration can be established by humans (also) with a variety of robotic agents, so that their performance is improved. The aim is to enhance the human capacity to solve cognitive tasks based on relations of social coordination with robotic artifacts, an empowerment often described through wide notions of the cognitive mind (e.g., [211]). Among these conceptual constructs, one of the most thought-provoking is the idea of a distributed mind, which defines a network—a co-evolutionary complex—of human and robotic agents that collaboratively coordinate their behaviors based on effective social signaling to generate (augmented) cognitive performance [212]. To maximize the success of these hybrid distributed minds—“cognitive (human-robot) ecologies”—social robotics grounds the believability of its social machines in synthetic modeling: the incorporation of scientific hypotheses about human social processes into these robotic artifacts and their behavioral patterns. This adds a significant scientific interest to social robots, making them synthetic—electromechanical—tools to deepen the scientific knowledge of human sociality based on a specific way of implementing the understanding-by-building method [213]. The scientific exploration of the quality of our social interactions with these robots allows specialists in social robotics to assess hypotheses on the human social processes that they embody in these machines and, in this sense, to positively contribute to the scientific understanding of our sociality. On these grounds, social robots have to be conceived of not only as artifacts to improve our daily lives but also, and inseparably, as tools serving our self-knowledge.

The application of SCs in the IoBNT offers to social robotics an unprecedented opportunity to extend these new lines of development, expanding them into the chemical domain. The new scenario that emerges today is that of *chemical social robotics*, engaged in the creation of synthetic and hybrid nano-cognitive ecologies grounded in effective forms of signaling—communication. The wide range of potential and incipient applications of SCs in the IoBNT, as described in this article, expresses the emerging options for the vast, programmatic work of developing new or augmented technological functionalities based on the design of forms of chemical communication that, by modeling minimal natural signaling processes, can generate productive cooperation between (artificial or artificial and natural) minimal interactive systems. However, as in the case of *electromechanical social robotics*, the ambition is not limited to the technological aim of building nanonetworks, fully synthetic or hybrid, that can increase our capabilities of performing cognitive tasks, enhance natural functions, or even disrupt processes, with negative impacts such as quorum sensing or pollution. What the cross-fertilization between SCs and the IoBNT opens is the possibility of exploring, at the nano-level, natural cognition (see Section 6.2) and, more specifically, social cognition based on a new application of the understanding-by-building method. The range of potential scientific developments is broad. They include deepening our understanding of sociality by synthetically reconstructing and studying some of its aspects, such as the nano-mechanisms involved in its biological and human forms, and segments of its natural paths of evolution, as well as exploring the transition from non-cognitive to minimally cognitive processes and from non-social to minimally social processes by attempting to create nano-forms of distributed organizations capable of accomplishing cognitive tasks based on behavior coordination grounded in effective signaling. Here, we present these prospective developments, recognizing that, as in *electromechanical social robotics*, *chemical social robotics* also requires an active effort to orient its development toward sustainability. And we believe that addressing this challenge involves the constitution of partnerships—communication—between disciplines, aiming to create a positive interplay between the processes of transformation and the processes of the generation of knowledge that these new techno-scientific developments can—and are starting to—produce.

## 10. Concluding Remarks

The IoBNT is a scenario, inspired by the IoT, consisting of an ecosystem of microscale or nanoscale devices that communicate with each other and with biological entities (cell, tissues, organs, …). It is possible to imagine that these devices will be designed and constructed by SB, possibly including microelectronic devices in the network, too. With recent advancements in MC [5,6,8], theoretical investigations directly or indirectly related to the IoBNT scenario are progressing reasonably well, allowing the identification of potentialities, providing an understanding of limitations and challenges, and devising essential architectures. On the other hand, experimental research probably lies a step behind. In this contribution, we have focused our attention on one particular SB tool that we believe can effectively contribute to the IoBNT: SCs constructed by bottom-up approaches. Their construction and features have been briefly presented.

MC is a promising technology to realize the IoBNT. Our discussion focused on the SC technology based on the exchange of chemical signals, which matches the MC philosophy perfectly. Indeed, several types of communicating SCs have already been constructed, showing communication among SCs or between SCs and biological cells, but the route toward mature communication engineering in these wetware systems is still at the beginning stage. The use of bottom-up SCs in an IoBNT scenario is therefore a challenging goal because of the rather simple and functionally limited structures it is possible to build with this approach (when compared to other sort of SCs). Consequently, the discussion remains, to a large extent, speculative at this stage, and here, we have just briefly sketched somewhat obvious IoBNT (or, sometimes, pre-IoBNT) scenarios. Our goal, in this respect, is more oriented toward eliciting interest among specialists in the MC and SB fields, calling for concrete attempts at collaboration. In other words, this contribution intends to be a call to action for all scholars who are interested in these exciting challenges, looking at future technologies from the viewpoint of the IoBNT.

If, on the one hand, this attempt (this perspective article) may appear too preliminary, it also represents an unprecedented opportunity to raise a new multi-, inter-, and transdisciplinary field, whose relevance for the science of the coming decades is clearly predictable. The synergy we identify will be brought to IoBNT applications. For example, it is important that MC scholars know more about the realistic possibilities of implementing SCs, currently or in the mid-term. In turn, well-calibrated MC theories can guide SB efforts so as to devise systems that channel the information flow in a proper manner and achieve the best performance for a certain pre-determined goal. The space for improvement and development appears quite wide. While current SCs still limp toward the goal of minimal communicative abilities, we are convinced that future developments (including neuromorphic engineering) have the potentiality, when jointly expressed with MC, of devising systems whose capabilities can be complementary to biological cells and wisely combined to generate original behaviors emerging from artificial/natural integration. The so-generated hybrid systems could have capacities that go beyond the capacities of living and SCs taken separately. Of course, new ethical issues are associated with these perspectives, as happens in many frontier technologies. The attitude of the SB community to consider and discuss the ethical aspects of its own research [214,215], as well as safety and security [216], provides a favorable starting point to also continue on this track for IoBNT applications. While current bottom-up SCs—due to their simple structures—are generally not considered in bioethical discourses, scenarios like the smart drug delivery application outlined in Section 7 will require, when realized, strict control of the safety of these sorts of new systems, which are more complex than the usual liposomes for drug delivery [217,218] but much less complex than any biological living cell, natural or engineered. In an authentic IoBNT scenario, artificial entities must interact with biological ones, with the former being specifically designed “to speak the same language” as the latter. This is per se a scenario that—in addition to the peculiar scientific interest (Section 9)—will deserve the attention of experts in biotechnological safety.

## Figures and Tables

**Table 1:** General characteristics of MC when compared to conventional telecommunications (for a more extensive/detailed table with comments, see [5]).

Feature	Telecommunication	Molecular Communication
Signal	Electromagnetic	Chemical
Speed	Fast	Slow
Distance	Long	Short
Media	Air, cables	Aqueous (mainly)
Transmission	Accurate	Stochastic

**Table 2:** Different scopes of SCs (often overlapping each other).

Research Area	Scopes
Origin of Life, Systems Chemistry	Building models of primitive cells (protocells) in order to shed light on the non-life-to-life transition; investigating cell-like structures made of primitive chemicals and functioning by primitive mechanisms; determining the minimal complexity of life; identifying the essential components and processes for the onset of life on Earth; understanding the emergence of life; investigating fundamental mechanisms, such as out-of-equilibrium dynamics, self-replication and self-reproduction, oscillations, confinement, nonlinearity, etc.
Sciences of the Artificial, Artificial Life, Theoretical Biology	Studying autonomy, agency, autopoiesis, self-organization, complexity, evolution, emergence of meaning, biosemiotics, etc., by means of physical or virtual cell models; understanding and comparing wetware models of life and cognition to hardware and software models (robotics and AI); exploring chemical AI, embodied AI, chemical robotics, autonomy, agency, communication and information theories, unconventional computing, cognition, enaction, embodiment, emergence, etc.
Molecular Communications, IoBNT	Building SCs as bio-nanoelements (devices) operating in bio-nanonetworks via molecular communication; engineering manipulation and transfer of chemical information; investigating communication and information theories, cybernetic aspects of molecular systems and syntactic and semantic information; constructing systems with sensing, control, actuation abilities, etc.
Biochemistry, Biophysics, Synthetic Biology, Biotechnological Applications	Building and using cell-like systems as an experimental platform to investigate complex biochemical or biophysical phenomena (i.e., reconstructing the target phenomenon in a simplified, and thus easier to study, context); exploring possible uses in nanomedicine, smart drug delivery, biosensing, bioproduction, nanoreactors, enzyme immobilization, tissue engineering, bioassays, etc.

## Data Availability

No data are associated to this article.

## Abbreviations

The following abbreviations are used in this manuscript:

ICTs	Information and communication technologies
MC	Molecular communications
SB	Synthetic Biology
SC	Synthetic Cell
IoT	Internet of Things
IoBNT	Internet of Bio-Nano Things
